# Supplementary material for: Proportion of Ugandans with pre-pandemic SARS-CoV-2 cross-reactive CD4+ and CD8+ T-cell responses: A pilot study
Source: PLOS Glob Public Health. 2023 Aug 16;3(8):e0001566. doi: 10.1371/journal.pgph.0001566 (PMC10431628; doi:10.1371/journal.pgph.0001566)
Supplement: S1 Fig — (DOCX) [file pgph.0001566.s001.docx]

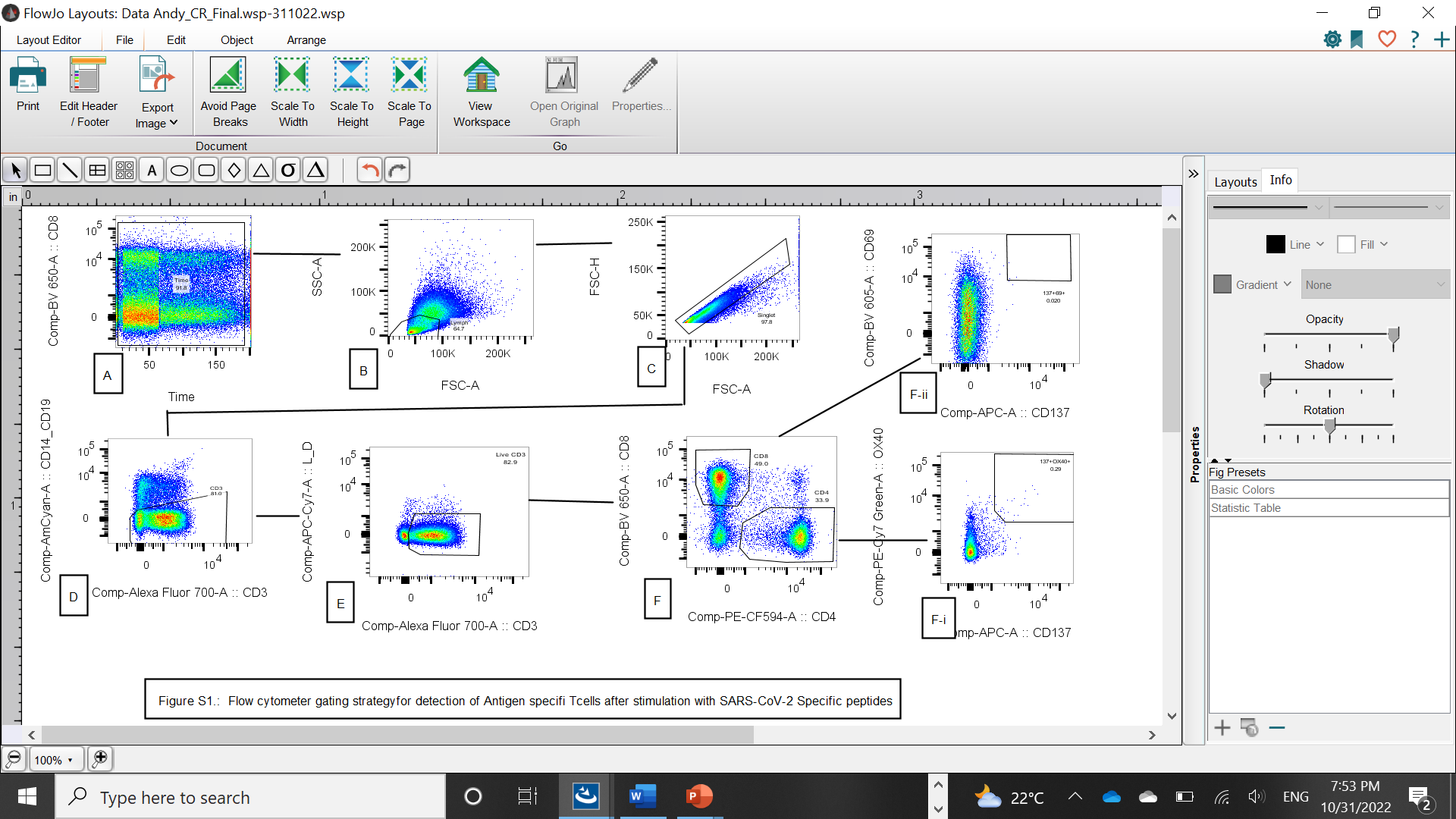
 **S1 Fig: Gating strategy (A-F) for detection of SARSD-CoV-2 reactive CD4+ and CD8+ cells after PBMC stimulation:** Time gating was done to eliminate any artifact like air bubble (**A**), followed by a selection of lymphocyte population (**B**), and singlets (**C**). Live CD3+ cells were selected (**D-E**), then divided into CD4+ and CD8+ cells (**F**). Within CD4 and CD8 subsets, antigen-specific T cells were established through the upregulation of activation-induced markers OX40, CD69, and CD137. Percentages of OX40+CD137+ double-positive cells within the CD4 gate (**F-i** ) and percentage of CD69+CD137+ within the CD8 gate ( **F-ii**) showing activated cells, were gated out to be used for further analysis
