# Supplementary material for: Proportion of Ugandans with pre-pandemic SARS-CoV-2 cross-reactive CD4+ and CD8+ T-cell responses: A pilot study
Source: PLOS Glob Public Health. 2023 Aug 16;3(8):e0001566. doi: 10.1371/journal.pgph.0001566 (PMC10431628; doi:10.1371/journal.pgph.0001566)
Supplement: S1 Table — (DOCX) [file pgph.0001566.s003.docx]

**Supplementary Table S1: AIM antibodies used for cell staining**

| **Membrane Antibody** | | **Fluorochrome** | **Clone/vendor/catalog** |
| --- | --- | --- | --- |
| 1 | CD45RA | BV421 | HI100/Biolegend/304130 |
| 2 | CD14 | V500 | M5E2/BD/561391 |
| 3 | CD19 | V500 | HIB19/BD/561121 |
| 4 | Live/Dead |  |  |
| 5 | CD8 | BV650 | RPA-T8/BioLegend/301042 |
| 6 | CD4 | PE-CF594 | RPA-T4RUO/BD/62316 |
| 8 | CCR7 | FITC | G043H7/Biolegend/353216 |
| 9 | CD69 | PE | FN50/BD/555531 |
| 10 | OX40 | PE-Cy7 | Ber-ACT35/Biolegend/350012 |
| 11 | CD137 | APC | 4B4-1/BioLegend/309810 |
| 12 | CD3 | AF700 | UCHT1/eBioscience/56-0038-42 |
