# Supplementary material for: Proportion of Ugandans with pre-pandemic SARS-CoV-2 cross-reactive CD4+ and CD8+ T-cell responses: A pilot study
Source: PLOS Glob Public Health. 2023 Aug 16;3(8):e0001566. doi: 10.1371/journal.pgph.0001566 (PMC10431628; doi:10.1371/journal.pgph.0001566)
Supplement: S2 Table — (DOCX) [file pgph.0001566.s004.docx]

**Supplementary Table S2:** Hospitalized COVID + samples

| COVID cohort, n=3 | CD4+ T cell Reactivity | % CD4+ Reactivity | CD8+ T cell Reactivity | % CD8 Reactivity |
| --- | --- | --- | --- | --- |
| **CD4_Non-spike** | 2 | 66.7% | 1 | 33.3% |
| **CD4_Spike** | 2 | 66.7% | 2 | 66.7% |
| **CD8_A** | 3 | 100.0% | 3 | 100.0% |
| **CD8_B** | 3 | 100.0% | 1 | 33.3% |
|  |  |  |  |  |
| **Total Pools Reactive** |  |  |  |  |
| **0** | 0 | 0.0% | 0 | 0.0% |
| **1** | 0 | 0.0% | 1 | 33.3% |
| **2** | 1 | 33.3% | 1 | 33.3% |
| **3** | 0 | 0.0% | 0 | 0.0% |
| **4** | 2 | 66.7% | 1 | 33.3% |
